# Dietary Exposure to Pesticide and Veterinary Drug Residues and Their Effects on Human Fertility and Embryo Development: A Global Overview

**DOI:** 10.3390/ijms25169116

**Published:** 2024-08-22

**Authors:** Ambra Colopi, Eugenia Guida, Silvia Cacciotti, Serena Fuda, Matteo Lampitto, Angelo Onorato, Alice Zucchi, Carmela Rita Balistreri, Paola Grimaldi, Marco Barchi

**Affiliations:** 1 Department of Biomedicine and Prevention, Faculty of Medicine and Surgery, University of Rome Tor Vergata, 00133 Rome, Italy; ambracolopi@yahoo.it (A.C.); eugenia.guida@uniroma2.it (E.G.); silviacacciotti1@gmail.com (S.C.); serena.fuda@gmail.com (S.F.); mlampitto@gmail.com (M.L.); onorato.ange@gmail.com (A.O.); azucchi97@gmail.com (A.Z.); p.grimaldi@med.uniroma2.it (P.G.); 2 Department of Biomedicine, Neuroscience and Advanced Diagnostics (Bi.N.D.), University of Palermo, 90134 Palermo, Italy; carmelarita.balistreri@unipa.it

**Keywords:** food contaminants, transgenerational inheritance, organophosphates, glyphosate, antibiotics

## Abstract

Drug residues that contaminate food and water represent a serious concern for human health. The major concerns regard the possible irrational use of these contaminants, since this might increase the amplitude of exposure. Multiple sources contribute to the overall exposure to contaminants, including agriculture, domestic use, personal, public and veterinary healthcare, increasing the possible origin of contamination. In this review, we focus on crop pesticides and veterinary drug residues because of their extensive use in modern agriculture and farming, which ensures food production and security for the ever-growing population around the world. We discuss crop pesticides and veterinary drug residues with respect to their worldwide distribution and impacts, with special attention on their harmful effects on human reproduction and embryo development, as well as their link to epigenetic alterations, leading to intergenerational and transgenerational diseases. Among the contaminants, the most commonly implicated in causing such disorders are organophosphates, glyphosate and antibiotics, with tetracyclines being the most frequently reported. This review highlights the importance of finding new management strategies for pesticides and veterinary drugs. Moreover, due to the still limited knowledge on inter- and transgenerational effects of these contaminants, we underlie the need to strengthen research in this field, so as to better clarify the specific effects of each contaminant and their long-term impact.

## 1. Introduction

**Figure 1:**
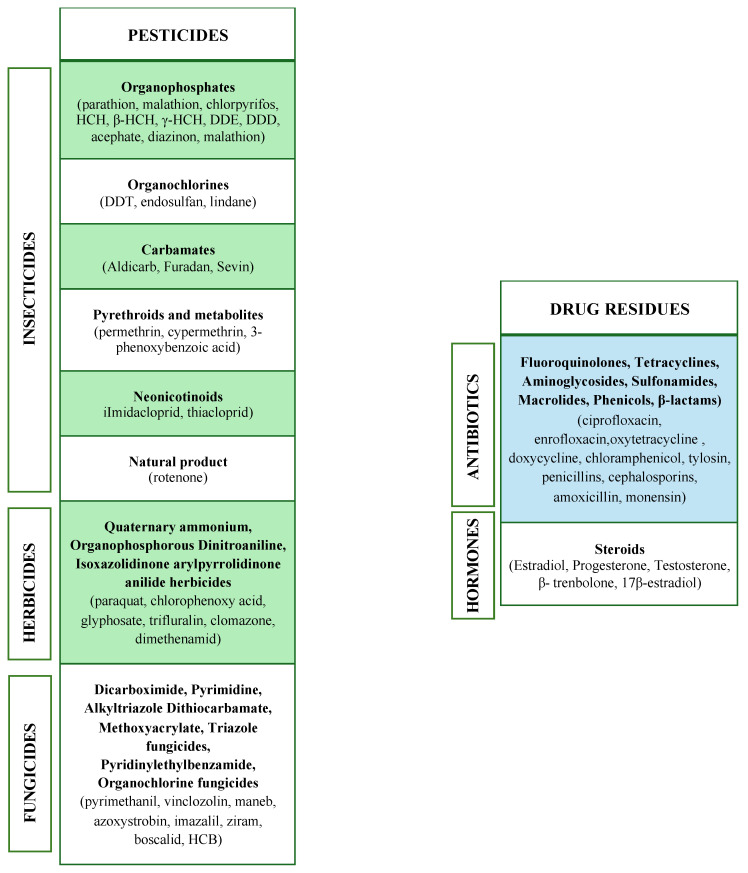
Pesticides and drug residues object of the study. Schematic representation of the classes of pesticide and drug residues analyzed in this review.

Anthropic activities have shaped the world, revolutionizing food production and transformation. Artificial “reinforcement” substances could be deemed necessary as additives or promoters in the food chain, to further enhance the yield for intensive animal farming and agro-industrial hyperproduction. Nevertheless, some of these substances have been proven to be harmful to the environment and plants, as well as to animals and humans. On top of that, the biggest challenge is represented by the indiscriminate use of these chemicals [1,2]. Several emerging contaminants, among which insecticides, herbicides and fungicides, microplastics, chemical ripening compounds, heavy metals, drug residues (antibiotics, hormones, anti-inflammatory drugs), personal care products, heavy metals, phthalates and per- and polyfluoroalkyl substances have been found to contaminate food, air and waters being potentially harmful for humans, animals and environment [3,4,5,6,7,8,9,10]. In this literature review, we focus on the possible toxic effects of pesticides and veterinary drug residues on human health (Figure 1).

Both pesticides and veterinary drugs, in fact, can be used in either rational or irrational ways. Rational use of veterinary drugs is intended when the animals receive medications appropriate to their clinical needs, with doses that meet the individual requirements and for a reasonable amount of time. Irrational drug uses, instead, are characterized by abuse, inappropriate dosage, prolonged duration of the treatment and unnecessary risk of drug resistance development. Similarly, pesticides are rationally employed when their application is spatially and temporally targeted. While on one hand, pesticides help farmers in intensively producing food, on the other hand, excessive use of pesticides results in contamination of surrounding soil and water sources, causing loss of biodiversity and are also proven to be harmful for farmers and consumers [11]. Thus, crop pesticide and veterinary drug residues contaminating food, air and water act synergistically in jeopardizing human health. These harmful compounds can be absorbed through several routes, including the respiratory and digestive tracts and accumulate in the body [12,13], potentially leading to detrimental effects on the health of young and adult individuals. Furthermore, the effect of these contaminants on fetal life is equally important.

Numerous pieces of evidence show a clear relationship between the exposure of pregnant women to toxic substances such as heavy metals, pesticides or other chemical pollutants (such as tobacco smoke, drugs, additives, organochlorines and air pollutants) and adverse reproductive and teratogenic effects [14]. However, data on the consequences of exposure to veterinary drug residues in the food chain for human offspring remain sporadic.

Therefore, more attention should be paid to their use and abuse.

With this literature review, we bring together recent knowledge on various toxic environmental contaminants (pesticides, herbicides, fungicides and drug residues), focusing on the consequences of human long-term exposure on health, with a special focus on fertility, embryo development and particularly on inter- and trans-generational effects. While the direct effects of these residues on human and wildlife health have been extensively described, their effects on the offspring of exposed parents are still scarcely understood. For these reasons, we aim to better clarify the mechanisms through which contaminants can influence inter- or trans-generational inheritance.

## 2. Food Contaminants of Agricultural and Veterinary Origin

Primary food contaminants arise from various sources, including agricultural use of pesticides and veterinary drugs.

### 2.1. Pesticides

The Food and Agriculture Organization (FAO) of the United Nations defines pesticides as “any substance or mixture of substances of chemical or biological ingredients intended to repel, destroy or control any pest, to improve plant growth” [15]. Generally, they can be classified by target life forms (e.g., herbicides, fungicides, insecticides, rodenticides), by chemical arrangement (e.g., natural, inorganic, engineered or organic) and by physical state (e.g., water solubility or volatility) [16]. Pesticide exposure results from multiple sources, including agriculture, primarily as insecticides (neonicotinoids, organophosphates, DDT, Pyrethroid, Rotenone), fungicides or herbicides (Glyphosate, Trifluralin, Paraquat). Moreover, contamination can further arise from domestic use, personal and public healthcare and vector control in specific areas [17].

As insecticides, neonicotinoids substitute many existing conventional insecticide classes due to their high efficiency, low resistance and less harmful effects on mammals. Simultaneously, due to their small molecular weight and high water solubility, neonicotinoids can penetrate plant tissues and be stored for a long time. These characteristics increase the probability of environmental contamination and exposure to nontarget organisms.

On the other hand, fungicides (e.g., Triazole, Maneb) are one of the core elements of intensive agriculture to fight pathogens that would cause large production losses [18]. Triazole fungicides (tebuconazole, triticonazole, hexaconazole, penconazole and uniconazole) have been classified as “potential human carcinogens” by the US Environmental Protection Agency and several studies demonstrate that these compounds can damage the kidneys of rats and pigs [19,20].

Glyphosate is the most common commercial synthetic phosphonate herbicide in the world [21]. It is intensively applied in crop fields and its residues are detected in the environment, particularly in plants, soil, water, food products and human urine [22].

### 2.2. Drug Residues

In addition to pesticides, another source of food contamination derives from residues of veterinary drugs. Useful to treat disease and improve animal health, veterinary drugs belong to different pharmacological categories, including antimicrobials and hormonal drugs. Several antimicrobial families are used in veterinary medicine: β-lactams (penicillins, cephalosporins, amoxicillin), sulphonamides (sulfadiazine, trimethoprim, sulfamethazine), tetracyclines, macrolides, aminoglycosides and quinolones (including fluoroquinolones, enrofloxacin, ciprofloxacin and ethoxyquin) [23]. Moreover, livestock drinking water is frequently supplemented with these drugs, representing one of the most economical routes of veterinary drug administration [24]. Penicillin has been extensively used in food-producing animals, particularly cattle, pigs and poultry, to control diseases and promote growth [25], while sulfonamides are commonly used in animal feed and fish cultures due to their high efficiency and relatively low cost [26].

The use of antibiotics in intensive animal farming can lead to the accumulation of these drugs in meat and other products such as milk and eggs. As reported by the EU statement, tetracyclines and sulfonamides are the most commonly used antibiotics in the pig industry, while tetracyclines (doxycycline) are used mainly in the poultry industry [27]. Furthermore, to obtain optimal profits and minimize costs and treatments, hormones are integrated in animal breeding to improve the rate of meat production on animal farms [28]. This class of veterinary drugs includes estradiol, progesterone, testosterone and bovine somatotropin [29].

Estradiol administration alone or in combination with progesterone or testosterone is used in order to increase rates of weight gain and feed efficiency in cattle [30], while bovine somatotropin is injected subcutaneously into dairy cattle and is approved as a method to increase milk production without affecting the micro- and macro-composition of milk [31].

Since drugs administered to animals and crops can volatilize, their residues can enter the human body through the food chain and also through inhalation [32], interfering with the endocrine system and disrupting physiological processes [33,34]. Despite the legislation, trace amounts of drug residues or their metabolites can be detected in air and animal-derived foods, causing potential adverse effects on human health, especially in the chronically exposed population [35].

## 3. Worldwide Distribution of Pesticides and Drugs Contaminants

The global distribution of pesticides and drug contaminants is an important environmental concern, affecting various ecosystems and human health. Studies have revealed widespread contamination across different regions, influenced by agricultural practices, industrial activities and regulatory frameworks.

### 3.1. Pesticides

The patterns of pesticide use vary greatly in different countries. Globally, around two million tons of pesticides are used annually, with the USA accounting for 24% of this total, Europe consuming 45% and the remaining 25% used in other parts of the world [36].

In 2021, the FDA pesticide residues report showed that no residues were found in aquatic products and fruits while both vegetables and grains products contained violative pesticide residues (9% and 11.8%, respectively) [37]. Among pesticides, the most frequently detected were imidacloprid, azoxystrobin and cypermethrin [37].

Spatial and temporal analyses conducted in Canada revealed significant pesticide occurrences (with chlorantraniliprole and various neonicotinoids having the highest frequency) in surface waters, with implications for aquatic ecosystems and human health [38].

In 2007, the Nordic Project analyzed samples of fruits and vegetables from different South American countries, including Brazil, Argentina and Chile. The results indicated that 8.4% of samples were above the Maximum Residue Limits (MRLs) and that thiabendazole, imazalil and chlorpyrifos were the most frequently found and detected with frequencies of 29.3%, 25.4% and 16.9%, respectively [39].

Among the countries of East Asia, China represents the one with the largest production and consumption of pesticides, resulting in serious pesticide pollution [40,41]. Particularly, pyrethroids were frequently detected in an active economic area that includes Guangdong (Southern China) and the Pearl River Basin [42]. Moreover, a recent study has demonstrated that different regions of China are still subject to pesticide risk, including Shandong, Henan and Hunan [43]. In particular, high detention rates of procymidone, lambda-cyhalothrin, cypermethrin, pendimethalin and isocarbophos were found in vegetable samples (27.0%, 16.2%, 11.4%, 3.5% and 1.9%, respectively) [44]. A study conducted in India revealed significant regional variations in pesticidal consumption. Key pesticide levels, including organophosphates, carbamates and synthetic pyrethroids, were considerably higher in the agricultural regions of Punjab and Haryana [36]. Analyses on food products including cereals, vegetables and fruits taken from both Delhi and Dehradun revealed that chlorpyrifos and chlorpropham were detected with values of 33% and 25%, respectively [45].

In 2022, a study reported European and Iceland and Norway levels of pesticide residues in foods, revealing that in 3.7% of samples, the MRL was higher than the legal limits. In particular, the pesticides with higher quantification rates were copper compounds (5.1%), ethylene oxide (2.3%) and chlordecone (1.0%) [46]. An analysis of chemical contaminants in the waters used for irrigation and livestock in Veneto region of Italy revealed high toxicity according to the growth inhibition test for *Pseudokirchneriella subcapitata*. Thus, from an ecotoxicological perspective, these waters cannot be deemed safe [47]. Furthermore, an evaluation conducted on aromatic herbs, collected from specific regions in Southern Italy, determined that herbicide residues exhibited substantial bioaccumulation [48]. Analyses on air, biota, water soil, sediment, vegetables and food products revealed that organochlorines (OCPs) are widely distributed in different environmental compartments in Africa, especially HCHs, DDTs and endosulfan [49]. Chromatography analyses on fruit and vegetables taken from Uganda detected pesticide residues in the 96,5% of samples, where organophosphates, carbamates, pyrethroids and neonicotinoids are most frequently found (91.3%, 67.5%, 60.0% and 42.5% respectively) [50].

### 3.2. Drug Residues

In addition to pesticides, the presence of pharmacological compounds, such as antibiotics and steroids, also poses a significant threat. As seen by a multitude of studies, the dispersion patterns of pharmaceutical contaminants exhibit significant variation across global regions.

The widespread presence of pharmaceutical drugs in our marine ecosystems and soil raises serious concerns, not only about their direct impact on the environment but also about their influence on the human food chain. An analysis by FAO and World Health Organization (WHO) has brought light to this issue, examining a range of veterinary drugs and assessing their presence in food. The findings revealed that many of the compounds analyzed are present in significant amounts, sometimes exceeding permissible limits for human consumption [51]. This implies that by eating foods originating from heavily polluted regions, we might ingest daily amounts of contaminants higher than what is considered safe for our health.

Veterinary drug residues were found to be excessively accumulated in animal products in South American countries including Argentina, Brazil and Uruguay. Ivermectin, doxycycline and monensin were detected in higher concentrations in animal food and their derivatives from Argentina and Uruguay [52]. Studies carried out in Europe on the Meuse river basin, which serves as a drinking water source to more than six million people, revealed the presence of a multicomponent snapshot of pharmaceuticals, such as antibiotics, analgesics and hormones and pesticides, overall providing insight on the intricate interaction between urbanization and industrialization and water quality [53]. The intricate dynamics of pharmaceutical contamination were further explored in the Guadalquivir river basin, in Southern Spain, highlighting the presence of drug compounds and their active metabolites in the waters [54]. Further research conducted in the United Kingdom delved into the environmental transport of antibiotics or analgesics to streams, elucidating the complex pathways and fate of these compounds in aquatic ecosystems [55].

A study of the aquaculture or wild-caught samples of pangasius (basa), cod, salmon, sole, tilapia, trout, white shrimp and giant tiger prawn originating from Canada, China, India, Southeast Asia (Indonesia, Thailand, Vietnam) and other regions worldwide revealed that 38% of the tested fish and shrimp samples had detectable residues of veterinary drugs or metabolites (among which were leucomalachite green, tetracyclines and metronidazole) and 25% were not compliant with Canadian guidelines [56].

Research on the aquatic environment of Vietnam highlighted that the occurrence of such contaminants was even higher than that found in international studies [57]. Furthermore, a study conducted in Shanghai, East China elucidated the occurrence and associated human health risks of pharmaceutical residues in the drinking water source, finding that all compounds of interest (ibuprofen, ketoprofen, naproxen, diclofenac and clofibric acid) were present in samples, with ketoprofen at the highest concentration and clofibric acid at the lowest [58]. Furthermore, investigations in North Indian cities underscored the prevalence of pharmaceuticals in urban wastewater, necessitating rigorous risk assessment protocols to safeguard public health [59]. A study conducted in Turkey revealed the presence of unpredictable drug residues such as anti-inflammatory drugs (mostly tolfenamic acid), quinolone, beta-lactam, aminoglycoside and polymyxin residues in breast milk from mothers, which may have a possible impact on maternal and infant health [60].

Several studies reported the exceeded levels of veterinary drug residues in different African countries, where tetracycline, oxytetracycline and penicillin were found frequently in animal products [61,62].

Overall, these studies emphasize the global ubiquity of pharmaceutical contaminants and pollutants. Moreover, since different countries set different levels of tolerance of contaminants, this raises concerns regarding the import/export of food products among countries, especially in cases where legal import sanitary procedures are not followed. For these reasons, the need is clear for the standardization of regulatory measures and legal limits to mitigate their adverse impacts on ecosystems and human health.

## 4. Routes of Exposure

**Figure 2:**
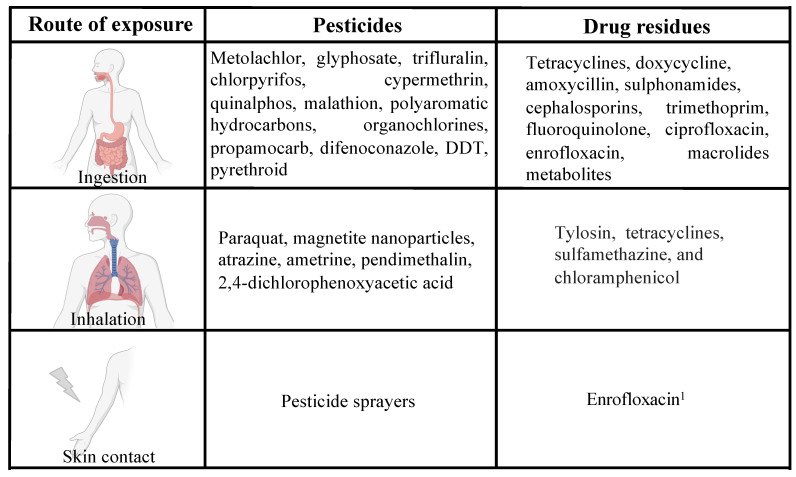
Pesticide and veterinary drugs routes of exposure. Exposure to environmental pollutants can occur through ingestion [65,66,67,68,69,70,71,72,73,74,75,76,77,78,79,80,81,82,83,84,85,86,87,88], inhalation [89,90,91,92,93] and skin contact [94,95,96,97,98]. For each route of exposure, examples of pesticide and veterinary residues have been reported. Created with BioRender.com. ^1^ possible, but negligible.

Humans can be indirectly exposed to potentially harmful chemical compounds in several ways. Pollutants and drug residues can be detected in all environmental compartments, both aquatic (well, surface, tap and wastewater) and non-aquatic (river sediment, soil and vegetables) [63,64]. Thus, potential routes of exposure to these compounds are ingesting contaminated food and water and dermal contact or inhalation [64] (Figure 2).

### 4.1. Oral Exposure

Pesticides can be found in farm animal feed and, consequently, in many animal-derived foods. A 2023 study detected the presence of herbicides, insecticides and fungicides in 62% of Austrian dairy cattle feed, including Metolachlor, which is largely used in the USA but not approved on the EU market by Regulation (EC) 1107/2009 due to its human carcinogenicity [99]. The levels of detected pesticides exceeded MRLs in approximately 20% of the samples [99]. Glyphosate, the most widely used herbicide worldwide, can be easily detected in water, rain and air [65] and it can also be found in food and livestock feed [66]. Soybean-fed cows are exposed to high amounts of glyphosate, which can be detected in the intestines and animal droppings and urine, but also in the liver, spleen, kidney and muscles [66,67]. Glyphosate residues were also found in poultry feed and within their eggs and their levels were above those imposed by MRLs [68]. High concentrations of glyphosate were also found in urine samples from horticulturists after the use of glyphosate-based pesticides and the values were significantly different from those observed in urine samples before the use of such compounds, indicating probably accidental ingestion during activity [69].

The use of chemical compounds such as pesticides, disinfectants, probiotics, feed additives and insecticides is also widespread in the aquaculture sector, as growth promoters or to prevent bacterial infections and the growth of algae or weed species and to improve water quality [70,71]. In addition, the same chemical agents used in agricultural soils or added to animal feed are dispersed in the environment, reaching seas, rivers and lakes [65] and can accumulate in fish and seafood tissues. For this reason, residues of pesticides (Trifluralin and chlorpyrifos, cypermethrin, quinalphos and Malathion) and chemical contaminants (polyaromatic hydrocarbons and organochlorines) were found in pangasius fillets from India and Vietnam [70] and traces of fungicides (Propamocarb and Difenoconazole) were detected in mantis shrimp [72]. Oral exposure to environmental contaminants can lead to the accumulation of such compounds in human tissues, so pesticide residues have been found in urine samples from agricultural workers and families residing in agricultural areas [73]. Furthermore, traces of DDT and Pyrethroid, two insecticides frequently used in Southern Africa to control malaria diffusion, were detected in most breast milk samples obtained from 152 mothers, with the highest concentrations found in primiparas [74], highlighting the problem of exposure to such pollutants even for newborns. The most common source of drug residues or pollutants in humans is drinking water or food. Pharmaceutical residues can also contaminate water sources, mainly from industrial effluents and runoff from agricultural activities or from the excretion of drugs by humans and animals. Thus, drinking water is also a considerable source of exposure to drug residues and pesticides for humans. Several studies identified the presence of antibiotic traces, hormones, parent compounds and degradation products in filtered tap water samples from different countries, pointing to possible concerns about their accumulation in humans [75,76,77,78,79,80]. Drug residues can be found in meat due to the use of antibiotics, hormones and other drugs in livestock farming and are transferred to humans through the consumption of meat or other animal-derived products, thus leading to the well-known phenomenon of biomagnification. The presence of pharmaceutical residues in food is the result of the frequent use of antibiotics, antiparasitic and non-steroidal anti-inflammatory drugs as feed and water additives in breeding centers. In fact, antibiotics are widely used to protect animals from diseases and infections and to implement the production of meat, dairy food and eggs. However, their increasingly intensive use can cause several critical issues, first of all, the development of antibiotic-resistant bacterial strains that can be transferred from animals to humans by ingesting meat or animal-derived foods [100], as has been reported for *Escherichia coli* and *Salmonella* species [101,102,103,104]. Although many eating habits are moving towards a plant-based diet, the consumption of meat and animal-derived products is constantly growing. Between 2022 and 2032, global meat production will increase by 12% and average per capita meat consumption will increase by 2% [105], while global egg and milk production will grow by 12% and 15% respectively [105] pointing to a growing risk of exposure to potentially harmful compounds in humans. To ensure consumer health, MRL for specific drugs have been established in animal-derived foodstuffs, considering the sum of the parent drug and its metabolites [81]. Among antibiotic classes, tetracyclines, doxycycline, amoxicillin, sulfonamides, cephalosporins and macrolides (particularly Tylosin) are commonly used as antibacterial in pig and chicken farms [82,83] and their metabolites are found within the intestinal tracts and in the feces of animals [84]. Antibiotic residues belonging to aminoglycoside, sulfamide, tetracyclines and macrolide classes were found in 32.39% of chicken meat samples from Algeria, with levels much higher than those imposed by MRLs [85]. Similar results were obtained when analyzing poultry meat samples from EU countries, with the prevalence of enrofloxacin and doxycycline [86]. The veterinary drug Sulfamethazine was found in eel samples along with the feed additive Ethoxyquin [87], which had been excluded from the EU list of allowed products in 2011 but then re-entered in 2017 [88]. Using Liquid Chromatography-Quadrupole High-Resolution Mass Spectrometry, residues of sulfadiazine, trimethoprim, fluoroquinolone, ciprofloxacin and enrofloxacin were identified in tilapia, catfish and shellfish [87].

### 4.2. Other Routes of Exposure

The most common route of exposure for pesticide-exposed workers, such as agricultural workers, urban pest controllers, municipal and park workers and foresters, is the dermal route. Spills and splashes can deposit on the worker’s skin during the mixing-loading phase and the pesticide application activity and can then be absorbed through the epidermis [94]. The absorption rate changes depending on the environmental conditions (temperature and humidity), the affected body part, the concentration of pesticides and the application of sunscreen, which can promote the penetration of pesticides through the skin [94]. It has been observed that the most affected body areas are legs and feet when using hand-held power sprayers and hands, arms and thighs when using manual sprayers [95,96]. However, the exposure intensity is 60% higher for manual sprayers than for hand-held power sprayers [96] and when using rear-mounted sprayers than when using trailer sprayers [97]. For this reason, the usage of personal protective equipment (PPE) [89] is strongly recommended for the occupational groups involved and ensures lower levels of dermal contamination when comparing the exposure of farmers wearing and not wearing protections [106], even if complete protection cannot be achieved [97]. By analyzing hand wipe samples, a recent study demonstrated that pesticide dermal contamination also occurs among families living near agricultural areas, although at much lower levels than those found among farmers’ families, both during the use period and the non-use period of such compounds [73,107,108]. Exposure to pesticides can also occur through inhalation of airborne aerosols and in this case, the most involved categories are those of agricultural sector workers [90]. Inhalation of toxic compounds offers a direct route to the brain through the olfactory nerves, therefore adverse effects on the central nervous system may occur. Several studies identified the potential risk of accidental exposure to airborne toxic compounds. Magnetite nanoparticles, for example, are commonly found in urban airborne particulate matter that can enter the brain directly through the olfactory nerve. Nanomagnetites can be potentially harmful because they are involved in the production of reactive oxygen species (ROS) and thus could be causally linked to neurodegenerative diseases [91]. A 2021 study exposed experimental mice to repeated low doses of Paraquat (PQ) aerosol to verify the presence of PQ residues in the brain, lungs and kidneys after exposure [90]. PQ is an herbicide that acts by damaging cells through the production of oxygen radicals and by compromising the photosynthesis process [109]. PQ was detected in all regions of the brain, with the highest concentration values found in the olfactory bulbs. However, the highest tissue concentration of PQ was observed in the lungs, while the lowest was observed in the kidneys. Furthermore, male mice showed a deficit in olfactory discrimination after exposure to PQ, indicating reduced olfactory function [90]. Another study analyzed 47 air samples obtained from workers responsible for applying pesticides at sugar cane farms in southern Africa. The presence of at least one of the four herbicides of interest (Atrazine, Ametryn, Pendimethalin and 2,4-dichlorophenoxyacetic acid) was detected in most samples, but Ametryn was detectable with the highest percentage rate (98.6% of the samples; [89]). A similar study was conducted in Malaysia by collecting 83 personal air samples from paddy farmers. The presence of the 13 targeted pesticides was observed in all samples, but none of the target compounds was associated with risks to human health derived from inhalation since their hazard quotient (HQ) was never lower than 1 [92].

Even if acute inhalation lethal concentration 50 (LC50) values have been established for these compounds, they may not consider adverse effects caused by exposure to lower but repeated concentrations. For these reasons, the concentrations of drug residues and pesticides that fall under the set threshold should also be carefully monitored.

The dermal route of veterinary drug residues is few reported. A study describing the pharmacokinetics of three uptake routes of enrofloxacin (dermal, oral and inhaled), revealed that the oral route was the major uptake route of enrofloxacin while dermal exposure was considered negligible for workers in hen houses [98].

Drug residues can be found in animal manure or be released in the air, becoming inhalable and causing respiratory diseases [8]. By analyzing dust samples from a pig house, a study revealed the presence of tylosin, tetracyclines, sulfamethazine and chloramphenicol with concentrations ranging from 0.2 to 12.5 mg/kg [93].

## 5. Impact of Pesticides and Drug Residues on Human Health at the Cellular Level

**Figure 3:**
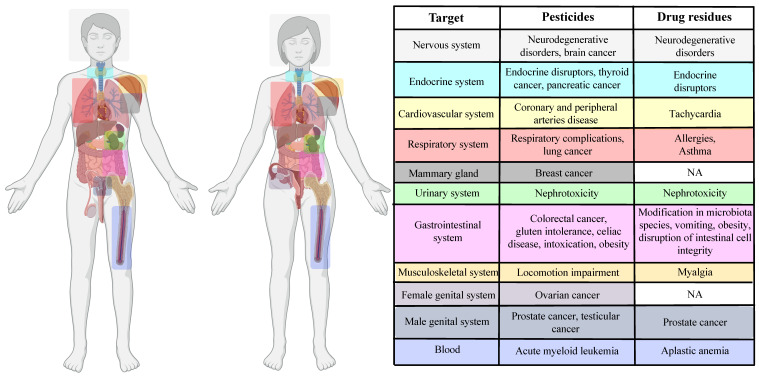
Effects of pesticide and drug residues on human health. Schematic representation of the main long-term effects of chemical and veterinary drug residues on human health [52,86,119,120,121,122,123,124,125,126,127,128,129,130,131,132,133,134,135,136,137,138,139,140,141,142,143,144,145,146,147,148,149,150,151,152,153,154]. Created with BioRender.com. Not assessed (NA) or no quantitative characterization.

Recent studies reveal that repeated exposure to drug residues and pesticides may lead to several pathological conditions [110], exposing humans to the increased risk of developing neurodegenerative disorders, endocrine disruptors, respiratory complications, reproductive disorders and birth defects and cancer [111,112,113,114,115,116,117,118] (Figure 3).

### 5.1. Pesticides

Numerous studies have suggested a link between pesticide exposure and cancer, among which glyphosate and acute myeloid leukemia (AML) or colorectal cancer (CRC) are the most studied ([119] and references therein).

Pesticides such as Paraquat and rotenone (analogues of 1-methyl-4-phenyl-1,2,3,6-tetrahydropyridine, MPTP) [120] and Maneb (a typical kind of dithiocarbamate (DTC) containing organic ligands and manganese ions, [121] increase the risk of developing Parkinson’s disease at an earlier age [122,123,124]. Exposure to such pesticides induces oxidative stress and reduces mitochondrial anterograde transport activity with subsequent damage to the neuronal synapse [122]. Studies in animals demonstrated that the brains of rotenone-treated mice underwent oxidative damage, mainly in the midbrain and olfactory bulbs [123]. This condition increases the formation of Lewy bodies and therefore the onset of Parkinson’s disease [122]. Maneb and its analog Mancozeb are also equipotent gastrointestinal toxicants proven to produce in vitro cell loss and metal overload, leading to oxidative stress [121].

Instead, pesticides such as malathion and parathion are considered risk factors for cancer diseases. Breast [125], thyroid [126], brain [119], colorectal [127], pancreas [119], lung [128], prostate [129] and ovary [130]. Studies on the effects of pesticides on the human microbiota demonstrated that serum levels of OCPs correlate with increased levels of methanobacteriales in the gut [131]. Moreover, methanobacteriales have been proven to be associated with obesity [132] and their concentration in the human gut has been linked to higher body weight and waist circumference [133].

Chlorpyrifos (CPF), a broad-spectrum organophosphate insecticide commonly used for pest control [134], has been found to be stored in adipose tissue, where it inhibits diet-induced thermogenesis and promotes obesity and insulin resistance [135]. In particular, brown adipose tissue of CPF-treated mice showed decreasing cAMP levels and the downstream signaling, highlighting a possible correlation between CFP and obesity [135]. Moreover, prolonged exposure to chlorpyrifos in mice can induce locomotion impairment and modify the characteristics of twitch contraction of skeletal muscle fibers [136]. A systematic review has reported a correlation between pesticides and cardiovascular diseases. It was found that organophosphates (OP) exposure is associated with coronary artery disease while OCP is linked with peripheral arterial disease [137]. The mechanism by which OP acts is in decreasing the paraoxonase (PON1) activity which low levels are correlated with coronary artery disease [138]. Among fungicides, triazoles have been shown to negatively interact with drug transporters in the human kidney, inducing nephrotoxicity [139]. Glyphosate has been shown to play a role in gluten intolerance, celiac disease and neurodegenerative disorders in humans [140].

### 5.2. Drug Residues

Animal meat and derivatives are well known to contain residues of drugs and antibiotics which, beyond a certain threshold, can be harmful to both humans and animals [52,85]. Drug resistance is the most feared risk for human health when considering antibiotic persistence food preparations from animal sources. It is estimated that 33,000 people die yearly because of selection of resistant bacteria to antibiotic therapies [155] and this phenomenon is due to the high exposure levels to antibiotics through the ingestion of overtreated animals [156]. Massive intake of food derived from animals treated with antibiotics could also lead to teratogenic effects [157] as well as to allergic and hypersensitivity reactions [86,141]. For this reason, some studies determined a limit value for each form of drug residual to establish an ingestion threshold that may not be harmful to our organism [156,158]. An example of drugs frequently used in livestock farming is ivermectin, an anthelmintic drug that, at high doses, can be toxic to humans, causing vomiting, tachycardia and myalgia [52]. Furthermore, the use of chloramphenicol in the zootechnical field has been banned in many countries around the world due to its strong toxic effects, such as the development of aplastic anemia [52]. Nevertheless, it is still illegally used in animal husbandry due to its effectiveness against infections caused by Gram-positive and Gram-negative bacteria and its residues have been found in cheese and meat [52].

Even if exposure to antibiotic residues through food is low, it can be assimilated to a long-term exposure. Unsupervised, long term-exposure to antibiotics has been widely demonstrated to induce antibiotic resistance [159] and represents a serious concern for human health. Several in vivo studies demonstrated that chronic exposure to antibiotics (such as tetracyclines, fosfomycin and doxycycline) can produce several side effects, including modification in microbiota species, decreased mucus secretion, reduction of digestive enzymes and disruption of intestinal cell integrity [142]. Exposure to residual doses of tylosin in early life has been associated with the development of metabolic disturbances by modifying the ratio of primary to secondary bile acids, thus exacerbating obesity [143]. Aminoglycocydes, instead, are known to cause adverse reactions such as nephrotoxicity and ototoxycity [144].

While no study has reported the effect of veterinary drugs on ovarian cancer development, recently it was found that 17β-trenbolone, which is used for rapid muscle development in cattle, induces the proliferation of prostate cancer cells [145]. In particular, 17β-trenbolone increases cell cycle-related proteins such as cyclin D2/CDK-4 and cyclin E/CDK-2, activating androgen-receptor [145]. Anticoccidial residues in food also exhibit toxic side effects, such as teratogenicity, hepatotoxicity or neurotoxicity, in laboratory animals treated with high doses of the drug [146].

The findings reported show the many possible side effects induced by pesticide and drug residues, highlighting the necessity of bioremediation strategies to minimize their impact on human health.

## 6. Effects of Pesticide and Drug Residues on Human Fertility, Embryo Development and Transgenerational Inheritance

**Figure 4:**
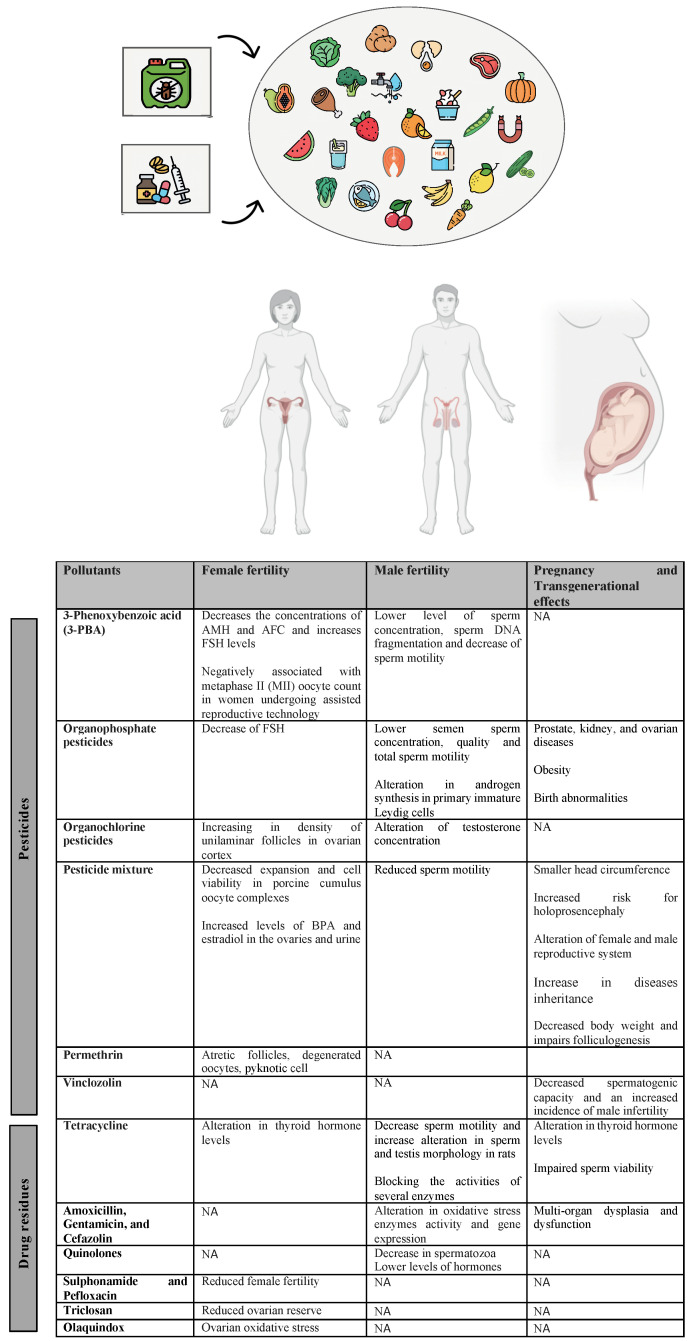
Pesticide and drug residues effects on female and male reproductive system and prenatal health. Exposure to environmental pollutants can occur mainly through the ingestion of contaminated animal-derived foods (upper figure). Once released into the body, polluting residues or their metabolites can alter human fertility and have adverse effects on pregnancy [113,149,154,171,172,173,174,175,176,177,178,179,180,181,182,183,184,185,186,187,188,189,190,191,192,193,194,195,196,197,198,199,200], as summarized in the table. Created with BioRender.com. Not assessed (NA) or no quantitative characterization.

Human fertility rates are decreasing worldwide [160]. The main causes of this decline include multiple factors such as social, educational, environmental and lifestyle factors [161]. Environmental and veterinary contaminants are negatively related to reproductive health [111,147] (Results are summarized in Figure 4). Pesticides mainly affect human reproduction by acting as endocrine disruptors [162], resulting in the increase or inhibition of endogenous hormones effects, or by inducing oxidative stress causing cell death and metabolic alteration in cells [163]. Exposure to both pesticides and drugs is also linked with epigenetic alterations, leading to intergenerational and transgenerational diseases and reproductive disorders [164].

### 6.1. Adverse Effects on Female Fertility and Pregnancy

The female reproductive system is strongly regulated by hormones that play a crucial role in the regulation of follicle growth and in the maintenance of reproductive function. During oogenesis, female germ cells give rise to primary oocytes through mitotic division. After its formation primary oocyte starts meiotic division and stops at prophase I. Oogenesis is tightly linked to folliculogenesis, a process in which granulosa cells proliferate and differentiate, establishing the maturation of the oocyte [165]. Germ cells and somatic cells of the ovary both can be affected by the endocrine disrupting activity of pesticides during the process of folliculogenesis and steroidogenesis [166,167].

#### 6.1.1. Pesticides

The main effects of exposure to pesticides in females include decreased fertility, spontaneous abortions, premature or low birth weight, developmental abnormalities, ovarian disorders and alteration of endocrine pathways [168]. Pyrethroids are pesticides commonly used in insect control in agricultural, residential and public sites [169]. According to the Agency for Toxic Substances and Disease Registry, human exposure to pyrethroids occurs by inhalation, ingestion, or dermal absorption, even if the rates of absorption through the lungs are not known and the dermal absorption appears to be minimal [170]. A study focused on analyzing the effects of permethrin (a pyrethroid insecticide) on rat ovaries showed that exposure to permethrin causes follicles atresia and oocytes degeneration. Pyknotic cells and condensed chromatin were observed in treated animals, indicating that the pyrethroid induced apoptosis of oocytes [171].

3-Phenoxybenzoic acid (3-PBA) has been detected in 66% of urine samples from women with a mean age of 33 years and has been shown to reduce the antral follicle count, decrease Anti-Müllerian Hormone concentration and increase follicle stimulating hormone (FSH) level, suggesting a potential impact on the ovarian follicle reserve [172]. The 3-PBA also affects the embryological outcome of women undergoing assisted reproductive technology by negatively impacting metaphase II oocyte count [173].

Among pesticides, OP, organochlorines and carbamates are considered the worst female fertility disruptors [148]. Exposure to organophosphate pesticides (OPPs) acts on menstrual cycles, sexual hormone imbalance and on ovarian weight [201]. A recent study reported the presence of several pesticide residues such as chlorpyrifos, diazinon, malathion and monocrotophos in blood samples from farm women aged 24 to 45 years, associated with alterations of FSH, LH (luteinizing hormone) and estradiol levels [174]. Similarly, exposure to dimethylphosphate has been found to correlate with female infertility in another cohort of women from the US, age ranged from 20 to 50 [175].

Comparable results have also been observed for other pesticide classes, such as OCP, proving a correlation between OCPs exposure and the density of unilaminar follicles in the ovarian cortex of American women [176].

Mixtures of pesticides could impact both folliculogenesis and steroidogenesis. It was found that a mixture of 10 different organochlorine pesticides including p,p′-DDT, p,p′-DDE, taxophene, αHCH, aldrin, dieldrin, 1,2,4,5-tetrachlorobenzene and lindane, decreased expansion and cell viability in porcine cumulus oocyte complexes compared to control [177]. On the other hand, the exposure to a mixture of triclosan, tetrabromobisphenol A (TBBPA), butyl paraben, propylparaben and DEHP causes the increasing of BPA levels and estradiol in the ovaries and urine respectively, suggesting that pesticides mixtures compete for enzymes in BPA and estrogen metabolic pathways [178].

Exposure of pregnant women to environmental contaminants is also a critical situation, for the potential risks on the offspring’s health and development. Several pieces of evidence highlight the correlation between exposure to pesticides during pregnancy and neurobehavioral deficits in the offspring. Prenatal exposure to OPP was reported to be associated with a smaller head circumference [179]. A case-control study demonstrated a two-fold increase in the risk of holoprosencephaly after exposure to personal insect repellents or pest control products during the preconception period and during pregnancy [113].

Prenatal exposure to pesticides can also affect birth body weight and the reproductive system of the offspring. Animal studies showed that daily exposure to a mixture of pesticides (composed of boscalid, captan, chlorpyrifos, thiacloprid, thiophanate and ziramthat) that can be commonly found in fruits in Europe causes offspring with decreased body weight and affects folliculogenesis [180]. Lindane was shown to induce both male and female fetal germ cell loss causing postnatal decrease of fertility in both sexes [181]. Exposure to OPP during gestation and lactation also affects the reproductive system of male offspring, leading to damage to testicular development and morphology. Although maternal exposure to OP was shown to not affect testicular function, male offspring were characterized by a decrease in the epithelium and the diameters of the seminiferous tubules and by an increase in the number of seminiferous tubules [202].

#### 6.1.2. Antibiotics

Although the adverse effects of most common veterinary drugs on human health are well established, their influence on the female reproductive system is poorly understood. The presence of sulfonamides and pefloxacin, used to prevent diseases in livestock production, in urine samples of female subjects, was associated with reduced female fertility [182]. Similarly, triclosan, an antibiotic used in cosmetics but also as a veterinary drug, has adverse effects on female fertility. It was found that urinary triclosan concentrations negatively correlate with antral follicles count, suggesting its possible effect on ovarian reserve [183]. Oxidative stress triggered by olaquindox, an antibacterial used to increase animal production, decreased the number of GV- and MII-stages oocytes and increased oocytes fragmentation and degeneration in mice [184]. Epidemiological studies revealed the presence of tetracycline in urine samples from pregnant women and correlated its levels with alteration of thyroid hormones which play an important role during pregnancy [149]. The adverse effect of amoxicillin exposure was found also in fetal development [185,203]. Mid or late pregnancy exposure to amoxicillin caused a decrease in body and tail length and body weight, as well as multiorgan dysplasia and dysfunction, which were sex related (males were more affected than female fetal mice) [185].

### 6.2. Adverse Effects on Male Fertility

#### 6.2.1. Pesticides

The effect of pesticides on male fertility is well documented. Several studies reported a correlation between 3-PBA metabolites in human residues and a lower level of sperm concentration, increased fragmentation of sperm DNA and decreased sperm motility [186,187,188]. However, Imai et al. reported that 3-PBA residues do not affect semen parameters such as sperm concentration and motility in young Japanese students [189]. A meta-analysis study showed that exposure to OP is negatively correlated with semen sperm concentration and total sperm motility compared to the unexposed group, while no significant correlation was found in serum concentrations of FSH, LH and testosterone in males [190]. Acephate, an organophosphate pesticide, affects androgen synthesis in primary immature Leydig cells from rats, blocking the transcription of several Leydig cell genes such as *Lhcgr*, *Star* and *Hsd3b1* [191]. Instead, DDT derivatives, including dieldrin and DDD, alter testosterone concentration in the serum of Chinese men with a median age of 30 [192].

Sperm chromatin is a sensitive target for OPP. Several lines of evidence show that male exposure to OPPs reduces sperm quality and total sperm counts [193] and interferes with proper male reproductive hormone levels by increasing FSH and luteinizing levels [193]. Among OPPs, γ-hexachlorocyclohexane (HCH), β-HCH, γ-HCH, 1,1-dichloro-2,2-bis (p-chlorophenyl) ethylene (DDE) and 1-dichloro-2,2,-bis (p-chlorophenyl) ethane (DDD) decrease sperm motility in concentration- and duration-dependent manners in vitro [194].

Studies performed mostly on rodents have shown that Lindane, HCB, α-Endosulfan, or PCB possess endocrine disrupting chemicals that can affect spermatogenesis, including testicular development and maturation during several critical stages of development, specifically in-utero development and puberty and can induce testicular cancer [150,151,152,153]. Mixtures of pesticides could negatively affect semen quality. Recent study demonstrated that both single or mixtures of pesticides negatively correlate with semen quality. In particular, it was shown that mixtures of pesticides reduce sperm motility and that Clomazone, Dimethenamid and Pyrimethanil exert the major effect among pesticides [195].

#### 6.2.2. Antibiotics

Due to their broad spectrum of activity, tetracyclines are the most used antibiotic compounds in the world [204,205]. Tetracyclines act primarily by inhibiting mitochondrial protein synthesis and this effect is particularly harmful to the male reproductive system [206]. Tetracyclines can decrease sperm motility and increase alteration of sperm and testes morphology enhancing oxidative stress by blocking the activities of several antioxidant enzymes such as superoxide dismutase, glucose-6-phosphate dehydrogenase and glutathione-S-transferase [196].

Oxidative stress induced by antibiotic residues may alter the expression of genes involved in male and female reproduction. Although exposure to amoxicillin, gentamicin or cefazolin did not significantly affect testes and cauda epididymis weights, decreased levels of glutathione but also increased levels of hydrogen peroxide were found in treated mice [197]. Oxidative stress in the testes was associated with an alteration in gene expression and the activity of antioxidant enzymes such as superoxide dismutase and catalase. Furthermore, the three antibiotics downregulated the expression of *Dazl* gene mRNA, which is essential for female and male germ cells [207,208], resulting in spermatogenesis failure [197]. The effects of ciprofloxacin (CIP) and enrofloxacin (ENR), which are quinolone antibiotics, were studied in zebrafish and mouse models [154]. Adult male zebrafish showed a significant decrease in spermatozoa after CIP treatment, suggesting its toxicity on spermatogenesis [154]. Moreover, CIP-treated zebrafish and ENR-exposed mice were characterized by lower levels of hormones (T, LH and FSH) and sperm compared to control groups, indicating that quinolone antibiotics could interfere with pituitary function [154].

## 7. Intergenerational and Transgenerational Effects

**Figure 5:**
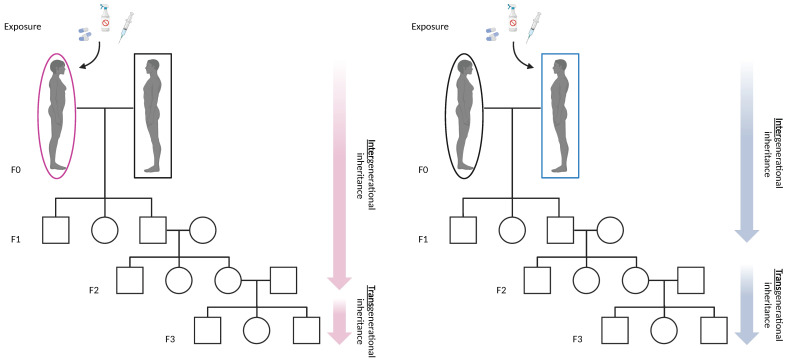
Intergenerational and transgenerational effects of environmental contaminants. Maternal and paternal exposure to pesticide and/or drug residue contaminants might increase the incidence of several pathologies in the offsprings through intergenerational and transgenerational epigenetic inheritance. Created with BioRender.com.

Exposure to environmental agents can induce epigenetic modifications in germ cells that can be passed to the progeny through successive generations causing intergenerational or transgenerational inheritance. Intergenerational inheritance generally occurs when a non-genetic modification is transmitted from exposed germ cells to their progeny. Conversely, transgenerational effects occur when germ cells not directly exposed to the environmental agents transfer the non-genetic modification to their progeny (Figure 5).

Starting from the environmental exposure, transgenerational inheritance occurs in the second-generation progeny, when males are exposed and at the third generation when females are exposed. The most studied epigenetic modifications induced by environmental cues are the epigenetic modifications, caused by small non-coding RNAs (sncRNAs), DNA methylation and histone modifications [209,210,211].

Several toxic substances have been identified to increase disease susceptibilities through intergenerational and transgenerational epigenetic inheritance. One of the first pesticides included in this category is vinclozolin, an agricultural fungicide used in the past in the vine industry [212]. Transient exposure to vinclozolin of a pregnant rat during gonadal sex determination induces in the F1 generation a decreased spermatogenic capacity and an increased incidence of male infertility. Interestingly, these effects are transferred through the male germ line to males of all subsequent generations up to the great–great grand offspring (F4) [213]. Based on the discoveries on vinclozolin, several other compounds have been tested for their possible transgenerational effects (reviewed in [214]). Glyphosate transient exposure of pregnant female rats can induce the transgenerational inheritance of several diseases that affect prostate (i.e., atrophic or hyperplastic prostate glandular epithelium), kidney (i.e., increased number of proteinaceous fluid filled cysts, reduction in size of glomeruli and thickening of Bowman’s capsules) and ovaries (i.e., polycystic ovaries), metabolism (i.e., obese phenotype) in F2 and F3 offsprings, while it possesses a negligible impact on the directly exposed F0 generation or F1 offspring. A delayed pubertal onset was also observed in males in the F1 and F2 generation. Testis disorders, instead, were characterized by the presence of azoospermia, atretic seminiferous tubules and lack of tubule lumen in F2 offsprings [198]. Pubertal abnormalities, testis and ovary disorders were observed in the progeny (F1-F3) of pregnant rats after the administration of the pesticide mixture composed of permethrin and N,N-diethyl-meta-toluamide (DEET), unraveling their transgenerational effect (on F3 offspring) in the inheritance of diseases [199]. Similarly, tetracyclines have also been demonstrated to induce an intergenerational effect on male reproductive function. In a study involving *Cordylochernes scorpioides,* males treated with tetracycline exhibited impaired sperm viability and this trait was transmitted to their male offspring (F1), but not to their grandsons (F2) [200]. Recently, the systemic impact of the administration of an antibiotic cocktail composed of neomycin, bacitracin and pimaricin on the gut microbiome and on the germline has been explored. Dysbiosis in fathers (achieved by the administration of antibiotics), increase the probability of their offspring presenting with low birth weight, severe growth restriction and premature mortality. Furthermore, gut microbiota perturbation in fathers causes downregulation of genes involved in placenta development, such as *Hand1* and *Syna*, as well as a reduction in placental growth factor (PLGF, a diagnostic marker of pre-eclampsia in humans [215] hormone levels and impaired vascularization. All these lines of evidence demonstrate that exposure to antibiotics has an impact on gut microbiome and on offspring fitness [216]. This study poses the potential risk of both voluntary and involuntary antibiotics administration on human health and on their inter- and trans-generational effects.

## 8. Concluding Remarks

Despite the fact that the legislation about the usage of pesticides in agricultural products and antibiotics in food-producing animals sets severe limits on tolerable maximum residues, trace amounts of drug residues or their metabolites may be still detected in several compartments; this is mostly due to the irrational use of these substances. The reasons that might contribute to this behavior are lack of information and sensibilization campaigns, inadequate education regarding good practices and increased food demand. Additionally, the expensive costs of veterinary services might induce farmers to engage in unsupervised treatment programs, thus using antibiotics and pesticides irresponsibly and without observing scheduled withdrawal periods. Moreover, the pervasive presence of these materials in water, soil, vegetable and animal-derived foods has been examined globally and it has been revealed that the regulatory measures currently undertaken cannot exclude the possibility of contamination, at least in traces. The list of emerging concerning compounds is constantly updated, as more studies are investigating their potential harm to humans and the ecosystem, revealing the necessity of following good practices to safeguard public health and the environment. For this reason, the extensive use of these pesticides and veterinary drugs, supported by the biomagnification process, invites stricter regulations and the promotion of sustainable agricultural practices to mitigate the adverse effects of the use of pesticides and veterinary drugs around the world. In addition, to maintain the fitness of the ecosystem, several bioremediation methods are currently available to eliminate harmful contaminants from the environment. Remediation strategies nowadays include phytoremediation, microalgae bioremediation, myco-remediation and bacterial pesticide degradation [217] and they represent a powerful method to help to minimize contaminant impacts on the ecosystem and on human health. Moreover, in the present review, we explored the many possible side effects of pesticide and drug residues on human health and in particular their effects on fertility and on inter- and trans-generational inheritance. Given the potential harmful behavior of the accumulation of these compounds in food, water, air and soil, their effect on human offspring should be further explored.
